# Analysis of Efflux Pump Contributions and Plasmid-Mediated Genetic Determinants in Ciprofloxacin-Resistant *Salmonella*

**DOI:** 10.3390/pathogens13121126

**Published:** 2024-12-20

**Authors:** Xiujuan Zhou, Shanrong Yi, Dai Kuang, Chunlei Shi, Chunbo Qu

**Affiliations:** 1 College of Public Health, Shanghai University of Medicine & Health Sciences, Shanghai 201318, China; 2 School of Agriculture & Biology, Shanghai Jiao Tong University, Shanghai 200240, Chinaclshi@sjtu.edu.cn (C.S.)

**Keywords:** *Salmonella*, ciprofloxacin, plasmid-mediated quinolone resistance, plasmid fusion

## Abstract

This study aimed to explore the interactions among genetic determinants influencing ciprofloxacin resistance in *Salmonella*. Treatment with PAβN, an efflux pump inhibitor, resulted in a 4–32-fold reduction in the minimum inhibitory concentration (MIC) across all 18 ciprofloxacin-resistant *Salmonella* isolates. Notably, isolates without point mutations reverted from resistance to sensitivity. The efflux pump played a crucial role in resistance development, particularly in serovar Enteritidis, where PAβN treatment caused a more significant MIC reduction (16–32-fold) in five strains carrying the GyrA (Asp87Tyr) mutation, which initially exhibited high MICs (8 μg/mL). Several resistance genes were identified on transferable plasmids: *oqxAB* and *aac(6′)-Ib-cr* were associated with IncF plasmids in *S.* Enteritidis, IncA/C plasmids in *S.* Typhimurium, and IncHI2 plasmids in *S.* Virchow. Additionally, *qnrS1* and/or *qepA* were carried by IncA/C plasmids in *S.* Thompson. Whole-genome sequencing revealed the presence of an *oqxAB* module integrated into the chromosomal DNA of *S.* Derby. Although the MICs of ciprofloxacin in transconjugants and transformants remained low (1–4 μg/mL), they exceeded the clinical breakpoint for susceptibility. These findings highlight the synergistic impact of efflux pumps and plasmid-mediated resistance mechanisms, contributing to the increasing prevalence of ciprofloxacin resistance and posing a significant threat to food safety.

## 1. Introduction

Salmonellosis is a significant global foodborne disease that affects both humans and animals. *Salmonella* serovars Typhimurium and Enteritidis are the most common causes of human nontyphoidal salmonellosis and are frequently associated with contaminated food products [1]. Additionally, surveillance data from China’s foodborne disease monitoring systems show an increasing rate of *Salmonella* isolation from patients with diarrhea [2]. Ciprofloxacin, a fluoroquinolone, is one of the first-line treatments for salmonellosis [3]; however, rising resistance to ciprofloxacin in *Salmonella* has become a major concern due to the misuse and overuse of antibiotics in humans and livestock. Recent reports indicate a growing prevalence of ciprofloxacin-resistant *Salmonella* in retail food products [4,5]. In 2017, the World Health Organization classified fluoroquinolone-resistant *Salmonella* as a high-priority pathogen posing a serious threat to public health [6], underscoring the challenges of effectively managing and controlling salmonellosis amid rising resistance to this critical antibiotic.

Ciprofloxacin resistance in *Salmonella* is primarily driven by point mutations in the quinolone resistance-determining regions (QRDRs) of the *gyrA* gene (encoding DNA gyrase subunit A) and the *parC* gene (encoding topoisomerase IV subunit C), which inhibit fluoroquinolones from binding to their target sites [7]. Additionally, overexpression of the AcrAB-TolC efflux pump, part of the resistance nodulation-division (RND) family, enhances fluoroquinolone resistance [8]. The AcrB multidrug transporter is particularly crucial for high-level fluoroquinolone resistance in some multidrug-resistant *S.* Typhimurium strains, where single QRDR mutations alone have minimal impact [9]. Moreover, several plasmid-mediated quinolone resistance (PMQR) genes, including *qnr*, *qepA*, *aac(6′)-Ib-cr*, and *oqxAB*, have been identified in *Salmonella* [10,11], contributing to both low-level fluoroquinolone resistance and the horizontal transfer of this resistance. The presence of these plasmid-mediated resistance genes suggests that bacteria carrying PMQR genes may possess a selective advantage; after surviving initial fluoroquinolone exposure, these bacteria could subsequently develop higher levels of chromosomal quinolone resistance over time [7].

Given the increasing concern over fluoroquinolone resistance, understanding the roles of PMQR genes and their interactions with QRDR mutations and efflux pumps in ciprofloxacin-resistant *Salmonella* is critical. In this study, 18 ciprofloxacin-resistant (CIP^R^) *Salmonella* strains were selected to assess the contributions of QRDR mutations, PMQR genes, and efflux pumps to ciprofloxacin resistance. The potential for plasmid transfer was evaluated using conjugation assays. Complete genome sequences of two representative CIP^R^ *Salmonella* strains, including their plasmids, were analyzed to investigate the transferable elements surrounding the quinolone resistance genes.

## 2. Materials and Methods

### 2.1. Ciprofloxacin-Resistant Salmonella Strains

A total of 18 CIP^R^ *Salmonella* strains were obtained in our previous studies [5,12], with mutations in the QRDRs and antimicrobial susceptibility testing for nalidixic acid being pre-determined (Table 1). To better explore the interplay between efflux pump activity and plasmid-mediated resistance mechanisms, we maintained a consistent genetic background in the strain selection, focusing on *S.* Typhimurium and *S.* Enteritidis, two major *Salmonella* serotypes. Specifically, six of seven *S.* Typhimurium and five of seven *S.* Enteritidis strains carried the *gyrA* mutation (Asp87Asn or Asp87Tyr), which are also the most prevalent in these two major *Salmonella* serotypes [5,12]. All the above eleven *Salmonella* strains, together with one *S.* Enteritidis strain without point mutations (SJTUF 13552), were isolated from food samples. The remaining four strains, which lacked QRDR mutations, included another *S.* Enteritidis (SJTUF 12553), *S.* Virchow (SJTUF 11258), and *S.* Derby (SJTUF 13534), all of which were clinical isolates. Additionally, a *parC* mutation (Thr57Ser) was detected in two clinical *S.* Thompson strains (SJTUF 12791 and SJTUF 11197). Antimicrobial susceptibility to ciprofloxacin with the concentration ranging from 0.125 μg/mL to 512 μg/mL was assessed using the agar dilution method, as recommended by the Clinical and Laboratory Standards Institute [13]. *E. coli* ATCC 25922 served as the quality control strain for minimum inhibitory concentration (MIC) determination, which had an MIC < 0.125, indicating susceptibility to ciprofloxacin.

### 2.2. Effects of Efflux Pump Inhibitor on Ciprofloxacin Resistance

Phe-Arg-β-naphthylamide dihydrochloride (PaβN) (Sigma-Aldrich, St. Louis, MO, USA) was used as an efflux pump inhibitor. Ciprofloxacin susceptibility was tested in parallel with and without PaβN. After the addition of ciprofloxacin and the bacterial inoculum, 2 μL of PaβN (5 mg/mL) was added to the microplate wells, bringing the total volume to 100 μL [14]. The remaining procedures for MIC determination followed the previously described protocol [13]. To ensure test accuracy and assess the effect of PaβN on bacterial growth, all strains were cultured in Mueller Hinton broth (Beijing Landbridge Technology Co., Ltd., Beijing, China) containing PaβN (100 µL/mL). All assays were performed in triplicate and repeated three times.

### 2.3. Plasmid Studies

Conjugation experiments were performed as previously described [15] using *E. coli* C600 as the recipient. Briefly, *Salmonella* isolates used as donors were incubated with the recipient overnight at 37 °C, and the overnight cultures were mixed and transferred to filter paper on a Luria–Bertani (LB) plate (Beijing Landbridge Technology Co., Ltd., China). Transconjugants were selected on MacConkey agar plates supplemented with ciprofloxacin (1 µg/mL) and rifampin (200 µg/mL). For the transformation experiments, plasmid DNA from CIP^R^ *Salmonella* was extracted using a Qiagen Plasmid Midi Kit according to the manufacturer’s instructions (Qiagen, Hilden, Germany). The purified plasmid was transformed into *E. coli* DH5α cells (Takara Biotechnology, Dalian, China). The transformants were selected on MacConkey agar supplemented with ciprofloxacin (1 µg/mL). Pulsed-field gel electrophoresis (PFGE) with S1 nuclease (Takara Biotechnology, Shanghai, China) digestion was performed to determine plasmid size. *S.* Braenderup H9812 was used as a DNA size marker. Plasmid incompatibility (Inc) groups were assigned using polymerase chain reaction (PCR)-based replicon typing with total DNA (including genomic DNA and plasmid DNA) samples of the 18 CIP^R^ *Salmonella* isolates and their transconjugants or transformants as templates. Each isolate was tested for the presence of 18 plasmid replicons using PCR assays as described previously [16]. The presence of PMQR genes (*qnrA*, *qnrB*, *qnrS*, *oqxA*, *oqxB*, *qepA*, and *aac(6′)-Ib-cr*) was determined using PCR as described previously [5,12]. All isolates positive for *aac(6′)-Ib-cr* and *qnrS* were sequenced to identify their gene subtypes.

### 2.4. Whole-Genome Sequencing and Data Analysis

*Salmonella* cells were transferred to LB broth and incubated overnight at 37 °C at 200 rpm. Total DNA was extracted from overnight cultures using a QIAamp DNA Mini Kit (Qiagen, Hilden, Germany). Whole-genome sequencing (WGS) was performed by MajorBio (Shanghai, China) using a PacBio RS II system (Pacific Biosciences, Menlo Park, CA, USA). Briefly, a 10-kb DNA library was constructed and sequenced using single-molecule real-time sequencing technology. The PacBio RS II sequencing data were assembled using Canu software (v2.1) [17]), and quality control was performed with FastQC [18]. Genome annotation was carried out using RAST (http://rast.nmpdr.org, accessed on 6 March 2024) and BLAST (http://blast.ncbi.nlm.nih.gov/Blast.cgi, accessed on 9 March 2024). Open reading frames (ORFs) were identified with the ORF Finder program (http://www.ncbi.nlm.nih.gov/orffinder, accessed on 6 March 2024). The origin of transfer (*oriT*) in the DNA sequences of mobile genetic elements was identified using oriTfinder (https://tool2-mml.sjtu.edu.cn/oriTfinder/oriTfinder.html, accessed on 6 March 2024). Plasmid types were determined using PlasmidFinder (https://cge.food.dtu.dk/services/PlasmidFinder/, accessed on 6 March 2024).

### 2.5. Nucleotide Sequence Accession Numbers

The complete sequences of *S.* Typhimurium SJTUF 13270, *S.* Derby SJTUF 13534, and their plasmids (pSJTUF 13270A, pSJTUF 13270B, and pSJTUF 13534) were deposited in the NCBI database under the accession number PRJNA1002226.

## 3. Results

### 3.1. Correlation Analysis of Molecular Basis for CIP^R^ Salmonella Isolates

The ciprofloxacin MICs of the 18 *Salmonella* strains after inhibition of the RND efflux pump were evaluated and compared with those of untreated CIP^R^ *Salmonella*. Overall, the resistance to ciprofloxacin in all test strains decreased 4–32-fold after treatment with the RND efflux pump inhibitor (Table 1). The results revealed that the impact of efflux pumps on ciprofloxacin resistance was linked to the presence or absence of point mutations in the QRDR. Specifically, after inhibition, the MICs of all five strains lacking point mutations shifted from resistant to sensitive, suggesting that the efflux pump contributed to low-level fluoroquinolone resistance during early low-dose exposure. Notably, *S.* Enteritidis showed a larger range of reduction (16 or 32-fold) following inhibition, including in five *S.* Enteritidis strains with a GyrA (Asp87Tyr) mutation and relatively high MICs (8 μg/mL), which also converted to a sensitive MIC after inhibition.

In contrast, the sensitive *E. coli* strain acquired partial or full ciprofloxacin resistance (ranging from 1/64 to equal to that of the original CIP^R^ *Salmonella*) following plasmid transfer. The MIC of ciprofloxacin in these transconjugants/transformants remained lower (1–4 μg/mL) but above the breakpoint level (Table 1). Interestingly, the contribution of PMQR genes to fluoroquinolone resistance was associated with QRDR point mutations. Plasmids played a more significant role in strains without QRDR mutations, with the MICs of five transconjugants reaching half or the full value of the corresponding *Salmonella* strains (Table 1). In the two *S.* Thompson strains (SJTUF 12791 and SJTUF 11197) with a ParC (Thr57Ser) mutation (Table 1), point mutations in the QRDR had the greatest effect on resistance (MIC 64 or 128 μg/mL), followed by the RND efflux pumps (8-fold reduction), while plasmids had a limited impact (only 1/32 or 1/64 of ciprofloxacin resistance was acquired).

### 3.2. Transferable Ciprofloxacin-Resistance Genes and Plasmids

**Figure 1:**
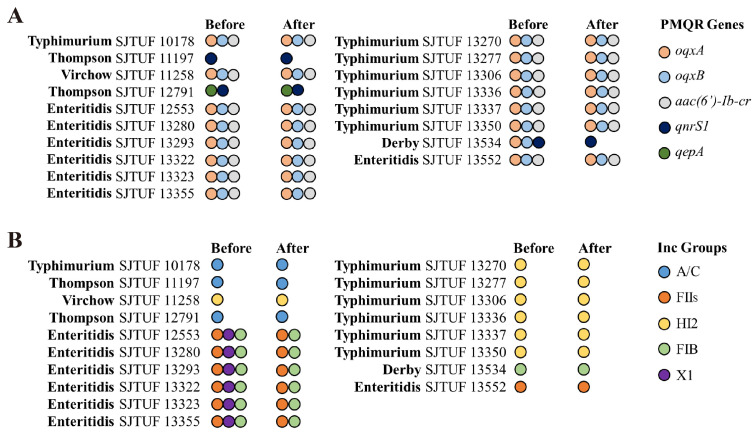
Comparison of *Salmonella* donors and the corresponding *E. coli* transconjugants/transformants based on plasmid-mediated quinolone-related genes (**A**) and plasmid incompatibly groups (**B**). Before, *Salmonella* donor; After, *E. coli* transconjugants/transformants. The plasmid-mediated quinolone resistance (PMQR) genes included *oqxA*, *oqxB*, *qepA*, *aac(6′)-Ib-cr*), and *qnrS1*, while plasmid incompatibility (Inc) groups included A/C, FIIs, HI2, FIB, and X1.

Since transferable plasmids were relevant to ciprofloxacin resistance, 7 known ciprofloxacin resistance genes and 18 common plasmid types were tested using PCR in both the original Salmonella strains and their corresponding transconjugant/transformants (Figure 1). Sixteen and 15 of the 18 CIPR isolates carried oqxA/B (88.9%) and aac(6′)-Ib-cr (83.3%) respectively, while 3 carried qnrS1 (16.7%), and 1 isolate carried qepA (5.6%) (Figure 1A). A total of 5 replicon types: FIIs, FIB, X1, HI2, and A/C, were detected in 18 CIPR isolates (Figure 1B); 6 *S.*. Enteritidis isolates carried Inc F plasmids (FIIs and FIB) and Inc X1 plasmids, and 6 of 7 *S.*. Typhimurium and 1 *S.* Virchow isolate carried IncHI2 plasmids; moreover, IncA/C plasmids were found in 2 *S.* Thompson and the remaining *S.* Typhimurium (SJTUF 10178) isolates, and *S.* Derby SJTUF 13534 harbored only the IncFIB plasmid, whereas *S.* Enteritidis 13522 contained only the IncFIIs plasmid.

In the conjugation experiments, all tested ciprofloxacin resistance genes in *S.* Enteritidis, *S.* Thompson, and *S.* Virchow, along with 1 *S.* Typhimurium strain carrying an Inc A/C plasmid, were transferable, with a conjugation efficiency of approximately 10^−6^. These genes were co-transferred with their corresponding plasmids (Figure 1). However, the ciprofloxacin resistance genes in 7 *S.* Typhimurium strains with IncHI2 plasmids were not transferable under the current conjugation conditions. Consequently, these IncHI2 plasmids were transformed into *E. coli* through electroporation. Interestingly, only *qnrS1* was found on the transferable FIB plasmid in *S.* Derby (SJTUF 13534) (Figure 1A), whereas *oqxA* and *oqxB* were absent, indicating that these two genes were likely located on the chromosome.

**Figure 2:**
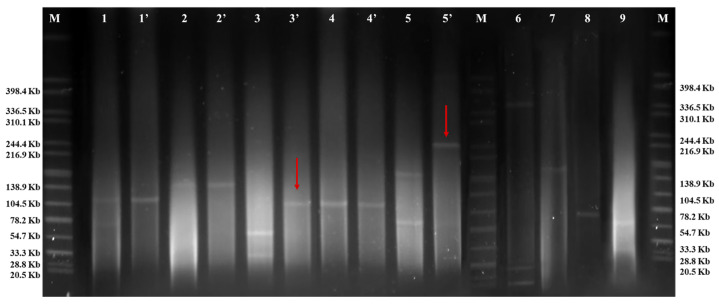
S1 nuclease pulsed-field gel electrophoresis of representative *Salmonella* donors (1–9) and corresponding *E. coli* transconjugants/transformants (1′–5′). M: H9812; 1: SJTUF 11197; 1′: SJTUF 11197-T; 2: SJTUF 12791; 2′: SJTUF 12791-T; 3: SJTUF 13280; 3′: SJTUF 13280-T; 4: SJTUF 13534; 4′: SJTUF 13534-T; 5: SJTUF 13270; 5′: SJTUF 13270-T; 6: SJTUF10178; 7: SJTUF11258; 8: SJTUF13552; 9: SJTUF 12553.

S1-PFGE was employed to determine the plasmid sizes in both the original *Salmonella* strains and their corresponding transconjugants/transformants. The S1-PFGE patterns of 9 representative original *Salmonella* strains and 5 representative transconjugants/transformants are shown in Figure 2. Five *S.* Enteritidis strains (SJTUF 13280, SJTUF 13322, SJTUF 13323, SJTUF 13293, and SJTUF 13355) shared identical S1-PFGE patterns, as did 6 *S.* Typhimurium strains (SJTUF 13270, SJTUF 13277, SJTUF 13306, SJTUF 13336, SJTUF 13337, and SJTUF 13350) and their transconjugants/transformants. Unfortunately, despite more than three tests being conducted, the S1-PFGE profiles of the four transformants (SJTUF10178-T; 7: SJTUF11258-T; 8: SJTUF13552-T; and 9: SJTUF12553-T) could not be obtained. Moreover, plasmid fusion occurred through plasmid co-conjugation in the 5 *S.* Enteritidis strains and through plasmid transformation in the 6 *S.* Typhimurium strains (Figure 2, 3′ and 5′, marked with red arrows), as the size of one plasmid in the transconjugants/transformants equaled the sum of the sizes of the two plasmids in the original *Salmonella* strains.

### 3.3. Complete Sequence of Representative Isolates and Transferable Element Analysis

**Figure 3:**
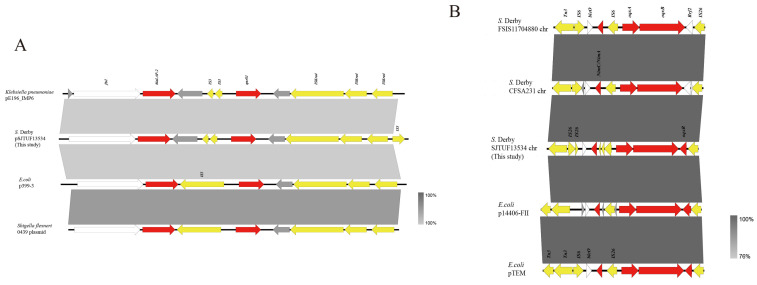
Analysis of the genetic environment of PMQR genes in strain SJTUF 13534. (**A**) Genetic environment comparison of *qnrS1* and *bla*_LAP-2_ genes in plasmid pSJTUF 13534, *K. pneumoniae* pE196_IMP6 (accession no. AP019405), *E. coli* p399-3 (accession no. CP084537), and *S. flexneri* 0439 plasmid (accession no. CP020344). (**B**) Genetic environment comparison of *oqxA* and *oqxB* genes in the chromosomes of SJTUF 13534, *S.* Derby FSIS11704880 (accession no. CP082411), and *Salmonella* CFSA231 (accession no. CP033350), and *E. coli* p14406-FII (accession no. MN823988) and *E. coli* pTEM (accession no. CP047003). Areas shaded in gray indicate homologies between the corresponding genetic loci on each plasmid. Boxes or arrows represent the open reading frames. Red, antibiotic resistance genes; yellow, insertion sequence/transposase; blue, replication-associated genes; gray, hypothetical protein; white, other genes.

WGS was performed on the isolate *S.* Derby SJTUF 13534 and its plasmid using the PacBio RS II system to analyze the surrounding genetic environment of the 3 PMQR genes (*qnrS1*, *oqxA* and *oqxB*). Figure 3A shows that a typical *qnrS1* resistance module structure (*fst1*-*bla*_LAP-2_-IS3-*qnrS1*-*orf1*-ISKra4-ISKra4-ISKra4) was identified in the 8.8-kb multidrug resistance region (MRR) in the FIB-type plasmid pSJTUF 13534, which showed a high similarity (~96%) to the MRRs of *K. pneumoniae* pE196_IMP6 (accession no. AP019405), *E. coli* p399-3 (accession no. CP084537), and *S. flexneri* 0439 plasmid (accession no. CP020344). It was further confirmed that the other 2 quinolone resistance genes (*oqxA* and *oqxB*) (Figure 1A) were located on the chromosome of *S.* Derby SJTUF 13534 (Figure 3B). The typical *oqxAB* module structure (IS26-*bleO*-NimC-IS26-*oqxA*-*oqxB*-Rrf2-IS26) in this study was also observed in other *Salmonella* chromosomes of *S.* Derby FSIS11704880 (accession no. CP082411) and *Salmonella* CFSA231 (accession no. CP033350) and was identical to that of p14406-FII (accession no. MN823988) and pTEM (accession no. CP047003) from *E. coli* (Figure 3B). The *oqxAB* module was likely transferred via IS26 mobilization between the plasmids and chromosomes in closely related Enterobacteriaceae (Figure 3B).

**Figure 4:**
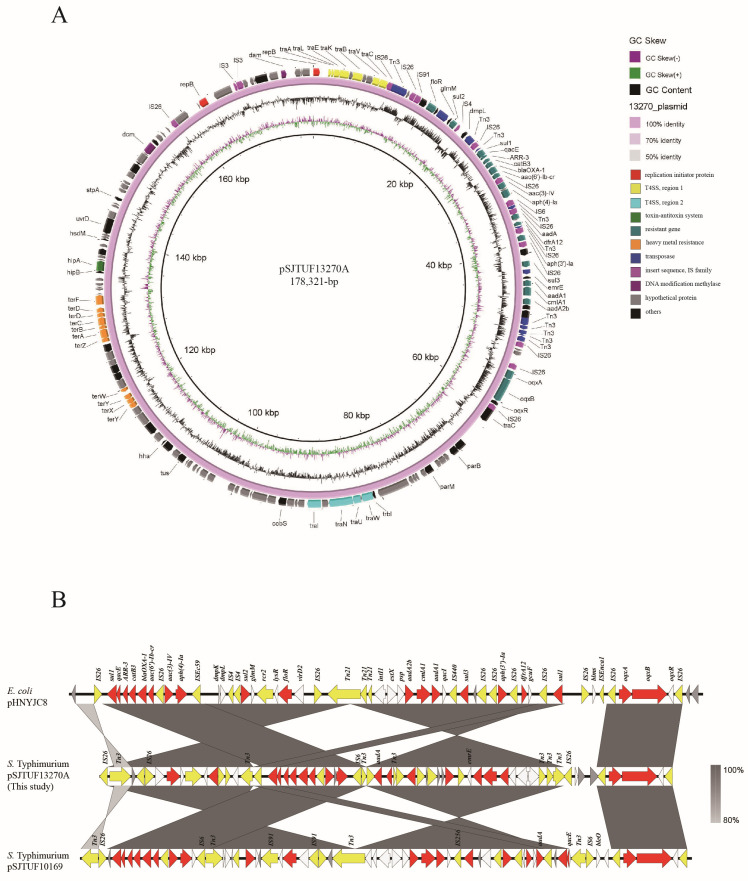
An overview of the plasmid pSJTUF 13270A (**A**) and its sequence comparison (**B**). The plasmid pSJTUF 10169 (accession no. CP047549) from *S.* Typhimurium, and *E. coli* pHDYJC8 (accession no. KY019259) were compared with the plasmid pSJTUF 13270A in this study. Areas shaded in gray indicate homology between the corresponding genetic loci on each plasmid. Boxes or arrows represent open reading frames. Red, antibiotic resistance genes; yellow, insertion sequence/transposase; blue, replication-associated genes; gray, hypothetical proteins; white, other genes.

To analyze the potential reasons for transfer defects and plasmid fusion of these *S.* Typhimurium strains carrying IncHI2 plasmids, we sequenced 1 representative isolate, SJTUF 13270. A total of 5,44,752-bp whole-genome DNA sequences with a GC content of 51.91% were identified in *S.* Typhimurium SJTUF 13270, including a 4,988,456-bp chromosome sequence and 2 plasmids. The larger plasmid, pSJTUF 13270A, was identified to be 178,321-bp in size and possessed 175 predicted coding sequences with a typical IncHI2 backbone, based on the results of PlasmidFinder (Figure 4A). Three PMQR genes (*oqxA*, *oqxB* and *aac(6′)-Ib-cr*) that mediate resistance to ciprofloxacin were identified in plasmid pSJTUF 13270A, consistent with the PCR results (Figure 2). Although 2 T4SS (genes encoding the bacterial type IV secretion system) were identified in this study (Figure 4A), the absence of the origin of the transfer site (*oriT*), relaxase genes, and the gene encoding type IV coupling protein in the conjugative region of the IncHI2 plasmid may result in the failure of conjugative transfer of pSJTUF 13270A. As shown in Figure 4B, plasmid pSJTUF 13270A harbors an approximately 51-kb MRR, which is a mosaic structure flanked by IS26 fragments. The *oqxA* and *oqxB* genes, bound by IS26 elements at both ends, were identical to those in pSJTUF 10169 (accession no. CP047549) and pHDYJC8 (accession no. KY019259) (Figure 4B). Notably, the typical *aac(6′)-Ib-cr* module (IS26-*sul1*-*qacE*-*arr-3*-*catB3*-*bla*_OXA_-*aac(6′)-Ib-cr*-IS26) was found in the same orientation in these three plasmids, but in pSJTUF 13270A, the IS26 element near the *sul1* gene was in the opposite orientation, leading to the reverse insertion of the *aac(6′)-Ib-cr* module within the MRR (Figure 4B). In addition, virulence genes (*hipA*) and heavy metal resistance genes (*terA*, *terB*, *terC*, *terD*, *terF*, *terZ*, *terX*, *terY*, and *terW*) were identified in pSJTUF 13270A (Figure 4A), implying their involvement with other hazards to food safety. Although no antibiotic resistance or virulence genes were found on the smaller plasmid (pSJTUF 13270B), the presence of integrase, recombinase, and recombination-associated proteins suggests a potential role in plasmid fusion with the IncHI2 plasmid.

## 4. Discussion

In our study, a significant decline in MIC ranging from 4–32-fold was observed in all 18 ciprofloxacin-resistant *Salmonella* isolates following treatment with an efflux pump inhibitor (Table 1). This finding aligns with previous research [8,19] that highlights the role of the RND efflux pump in mediating fluoroquinolone resistance in *Salmonella*. The RND efflux pump has long been considered a key player in the development of antimicrobial resistance in *Salmonella* [20], particularly for fluoroquinolones. However, few studies have explored the impact of multiple efflux pumps across different *Salmonella* serotypes. Interestingly, in this study, we observed a more pronounced reduction (16-or 32-fold) in MIC values in the five *S.* Enteritidis isolates with higher baseline MICs (Table 1), underscoring the critical role of efflux pumps in fluoroquinolone resistance within this serovar. *S.* Enteritidis is recognized for its high genomic clonality, which may suggest that this serovar lacks alternative efflux pumps and regulators to compensate and regulate for the inhibition of RND pumps [21]. As a result, the inhibition of the RND efflux pump could be particularly effective in *S.* Enteritidis strains. This observation implies that efflux pump inhibitors may have greater therapeutic potential against bacteria with high clonality, such as *S.* Enteritidis. Further studies are needed to expand the validation of this approach, especially across a broader range of bacterial species and serovars, to fully explore the clinical applicability of efflux pump inhibitors as a treatment strategy.

Previous studies have shown that plasmids carrying PMQR genes can confer resistance levels above the ciprofloxacin breakpoint (≥1 µg/mL) in the absence of QRDR mutations, introducing a novel mechanism of fluoroquinolone resistance [22]. In our study, the MIC of ciprofloxacin in all transconjugants/transformants remained between 1–4 µg/mL, surpassing the breakpoint level (Table 1). It has been hypothesized that bacteria harboring PMQR genes may gain a selective advantage; after surviving initial exposure to fluoroquinolones, these bacteria may later develop high-level chromosomal resistance [7,23]. Essentially, once point mutations occur that confer high resistance, the impact of these plasmids becomes less pronounced. This molecular basis for ciprofloxacin-resistant *Salmonella* was evident in the two *S.* Thompson strains, where the plasmids had a limited impact (1/32 or 1/64 ciprofloxacin resistance was acquired) (Table 1). Therefore, plasmid-mediated transfer of PMQR genes likely represents a key risk factor for the rapid dissemination of fluoroquinolone resistance. Meanwhile, efflux pumps may assist bacterial adaptation to continuous antibiotic exposure, and QRDR mutations may drive the development of higher-level resistance.

The detection results of transferable ciprofloxacin-resistance genes in this study (Figure 1A) align with those of recent studies [12,24], where the most frequently identified PMQR determinants were *oqxAB* and *aac(6′)-Ib-cr*, with fewer detections of *qnr* and *qepA*. In our study, *oqxAB* and *aac(6′)-Ib-cr* were often co-located within a strain (15/18, 83.3%) (Figure 1A), suggesting that this co-occurrence may contribute to the increased incidence of ciprofloxacin resistance in *Salmonella* observed in recent years. Our findings from Inc group assays (Figure 1B) are consistent with previous reports [19,25,26] that *oqxAB* is frequently associated with IncF, IncX1, and IncHI2 plasmids. Notably, Shi et al. [27] further demonstrated that the spread of *oqxAB* and *aac(6′)-Ib-cr* in *S.* Typhimurium predominantly occurs via IncHI2 plasmids (Figure 4), which is consistent with the findings of our study. The *qnrS1* gene, typically associated with IncN plasmids in Enterobacteriales, including *Salmonella* [5,28,29], was also detected on Inc A/C plasmids in *E. coli* [30], similar to our finding where *qnrS1* was driven by Inc A/C plasmids in *S.* Thompson (Figure 1B). The broad host range of Inc C plasmids has been implicated in the co-dissemination of various antibiotic resistance genes [31,32], and recent reports from China identified *S.* Thompson isolates harboring both *qnrS1* and *qepA* on Inc C plasmids [33], a finding consistent with strain SJTUF 12791 in our study (Figure 1B). PMQR genes located on plasmids are commonly detected in CIP^R^ *Salmonella* strains across various serotypes [21,34,35]. These PMQR-carrying plasmids have been a driving force behind the sharp rise in CIP^R^ *Salmonella* prevalence over the past decade. One possible explanation for this rapid dissemination is that mobile genetic elements allow *Salmonella* to acquire ciprofloxacin resistance without incurring a significant fitness cost.

Recently, a new mechanism of ciprofloxacin resistance was reported, in which a combination of *qnrS1* and other PMQR genes located on a chromosomal fragment or plasmid was mainly observed in *S.* Derby as a result of clonal spread [12,36]. Further, a recent report on *S.* Derby [37] showed that this chromosomal DNA fragment flanked by IS26 could form a circular intermediate that facilitates the transmission of this DNA fragment between different *Salmonella* strains. The PCR and sequencing results of *S.* Derby SJTUF 13534 in this study (Figure 1A and Figure 3B) also verified the transmission possibility of the circular intermediate between different strains. Further studies are required to elucidate the mechanism of transmission of this circular intermediate.

The HI2 plasmids in the six *S.* Typhimurium strains were not transferable, primarily due to the absence of essential elements in the conjugative region (Figure 4A). Similar transfer defects caused by the lack of these elements have been noted in other HI2-type plasmids in *Salmonella* [24]. Another phenomenon that needs to be pointed out is that the X1 plasmid in *S.* Enteritidis did not transfer successfully and was not related to the transfer of PMQR genes (Figure 1A). This suggests that the X1 plasmid may lack transfer elements or may be linked to other genetic traits, which warrants further investigation, possibly through sequencing.

Plasmid fusion in *Salmonella* has been frequently reported over the past decade [38,39,40] and plays a significant role in the accumulation of antibiotic-resistance-encoding genes in foodborne *Salmonella*, posing a considerable threat to food safety. Conjugative helper plasmids can fuse with non-conjugative PMQR-encoding plasmids to facilitate the transmission of resistance genes among *Salmonella* strains [38,40]. Therefore, the fusion phenomenon observed in this study, particularly involving the smaller plasmid (pSJTUF 13270B) fusing with the HI2 plasmid, warrants serious attention. This is especially pertinent given the detection of integrase, recombinase, and recombination-associated proteins in pSJTUF 13270B (Figure 4A), which may enhance the mobility and dissemination of resistance traits within the bacterial population.

A limitation of our study is the focus on a specific subset of ciprofloxacin-resistant *Salmonella* strains, primarily from *S.* Typhimurium and *S.* Enteritidis, with repetitive mutations in the *gyrA* gene (Asp87Asn and Asp87Tyr). While this selection allowed us to maintain consistency with our previous research and investigate the interplay between efflux pump activity and plasmid-mediated resistance mechanisms within a controlled genetic context, it may limit the generalizability of our findings, as the homogeneity of *gyrA* mutations could restrict applicability to other *Salmonella* serotypes or strains with different resistance mechanisms. Additionally, by focusing solely on ciprofloxacin, the study did not explore the effects of other quinolones. Future research should include a broader range of strains and antibiotics to enhance the generalizability and comprehensiveness of the findings.

## 5. Conclusions

Our study highlights the crucial roles of efflux pumps and transferable plasmids in the mechanisms of ciprofloxacin resistance among *Salmonella* isolates. The marked reduction in MICs following the inhibition of RND efflux pumps emphasizes their contribution to fluoroquinolone resistance, particularly in *S.* Enteritidis. Furthermore, the identification of key PMQR genes across diverse plasmid types underscores the complex interplay between plasmids and chromosomal mutations in conferring antibiotic resistance. Notably, the phenomenon of plasmid fusion signifies a potential pathway for the rapid dissemination of resistance genes among *Salmonella* strains. Both efflux pumps and PMQR genes located on chromosomal fragments or plasmids are essential for fluoroquinolone resistance, with their effects linked to the presence or absence of point mutations in QRDR and variations among *Salmonella* serotypes. The diversity of plasmid types and the presence of circular intermediates facilitate the transfer of PMQR genes, further heightening the risk of resistance dissemination. This study emphasizes the necessity for ongoing surveillance of antibiotic resistance mechanisms in *Salmonella* and highlights the importance of addressing plasmid-mediated resistance in strategies to combat foodborne infections. Further research is essential to elucidate the mechanisms behind plasmid transfer and fusion, as these processes significantly contribute to the evolution of antibiotic resistance in bacterial populations.

## Figures and Tables

**Table 1:** Comparison of the MIC of ciprofloxacin in 18 ciprofloxacin-resistant (CIP^R^) *Salmonella* strains before and after the inhibition of resistance nodulation-division efflux pumps and transconjugant/transformant in *E. coli* C600/DH5α.

Strains	Serovars	QRDR	Nalidixic Acid	^a^ MIC of Ciprofloxacin (μg/mL)			Fold Changes *	
				Untreated *Salmonella*	Treatment with Inhibitor	^b^ *E. coli* Transconjugants/Transformants	Inhibition	Transconjugant/Transformant
SJTUF 13270	Typhimurium	GyrA (Asp87Asn)	R	16	2	4	8	1/4
SJTUF 13306	Typhimurium	GyrA (Asp87Asn)	R	16	2	4	8	1/4
SJTUF 13336	Typhimurium	GyrA (Asp87Asn)	R	8	1	2	8	1/4
SJTUF 13337	Typhimurium	GyrA (Asp87Asn)	R	16	2	4	8	1/4
SJTUF 13350	Typhimurium	GyrA (Asp87Asn)	R	16	2	4	8	1/4
SJTUF 13277	Typhimurium	GyrA (Asp87Asn)	R	8	1	2	8	1/4
SJTUF 10178	Typhimurium	NA	S	2	0.5	1	4	1/2
SJTUF 13552	Enteritidis	NA	S	1	0.03	1	32	1
SJTUF 13323	Enteritidis	GyrA (Asp87Tyr)	R	8	0.25	2	32	1/4
SJTUF 13293	Enteritidis	GyrA (Asp87Tyr)	R	8	0.25	2	32	1/4
SJTUF 13280	Enteritidis	GyrA (Asp87Tyr)	R	8	0.25	2	32	1/4
SJTUF 13322	Enteritidis	GyrA (Asp87Tyr)	R	8	0.5	2	16	1/4
SJTUF 13355	Enteritidis	GyrA (Asp87Tyr)	R	8	0.25	2	32	1/4
SJTUF 12553	Enteritidis	NA	S	1	0.06	1	16	1
SJTUF 12791	Thompson	ParC (Thr57Ser)	S	128	16	2	8	1/64
SJTUF 11197	Thompson	ParC (Thr57Ser)	S	64	8	2	8	1/32
SJTUF 11258	Virchow	NA	S	2	0.125	1	16	1/2
SJTUF 13534	Derby	NA	S	2	0.25	2	8	1

^a^, The MIC breakpoint of ciprofloxacin resistance is 1 μg/mL; ^b^, MIC of recipient *E. coli* C600 or *E. coli* DH5α was <0.125, which was susceptible to ciprofloxacin. *, Fold change for inhibitor = MIC in untreated Salmonella/MIC after treatment with inhibitor; fold change for transconjugant/transformant = MIC in *E. coli* transconjugants or transformants/MIC in untreated *Salmonella*. GyrA, gyrase subunit A; MIC, minimum inhibitory concentration; NA, not applicable; ParC, topoisomerase IV subunit C; QRDR, quinolone resistance determination region.

## Data Availability

The complete sequences of S. Typhimurium SJTUF 13270, S. Derby SJTUF 13534, and their plasmids (pSJTUF 13270A, pSJTUF 13270B, and pSJTUF 13534) were deposited in the NCBI database under the accession number PRJNA1002226.
